# Evolving Applications of Echocardiography in the Evaluation of Left Atrial and Right Ventricular Strain

**DOI:** 10.1007/s11886-024-02058-x

**Published:** 2024-04-22

**Authors:** Adam Serafin, Wojciech Kosmala, Thomas H. Marwick

**Affiliations:** 1 Wroclaw Medical University, Wroclaw, Poland; 2 Baker Heart and Diabetes Institute, Melbourne, Australia; 3 Present Address: Menzies Institute for Medical Research, Hobart, Australia

**Keywords:** Left atrium, Right ventricle, Myocardial deformation, Strain, Echocardiography

## Abstract

**Purpose of Review:**

Speckle-tracking echocardiography (STE) can assess myocardial motion in non-LV chambers—including assessment of left atrial (LA) and right ventricular (RV) strain. This review seeks to highlight the diagnostic, prognostic, and clinical significance of these parameters in heart failure, atrial fibrillation (AF), diastolic dysfunction, pulmonary hypertension (PH), tricuspid regurgitation, and heart transplant recipients.

**Recent Findings:**

Impaired LA strain reflects worse LV diastolic function in individuals with and without HF, and this is associated with decreased exercise capacity. Initiating treatments targeting these functional aspects may enhance exercise capacity and potentially prevent heart failure (HF). Impaired LA strain also identifies patients with a high risk of AF, and this recognition may lead to preventive strategies. Impaired RV strain has significant clinical and prognostic implications across various clinical scenarios, including HF, PH, tricuspid regurgitation, or in heart transplant recipients.

**Summary:**

STE should not be limited to the assessment of deformation of the LV myocardium. The use of LA and RV strain is supported by a substantial evidence base, and these parameters should be used more widely.

**Supplementary Information:**

The online version contains supplementary material available at 10.1007/s11886-024-02058-x.

## Introduction

Left ventricular (LV) strain is widely used in the detection of subclinical LV dysfunction (e.g., cancer treatment-related cardiac dysfunction), heart failure with preserved ejection fraction (HFpEF), the assessment of LV diastolic dysfunction, risk stratification in aortic stenosis and mitral regurgitation, and many other LV diseases [1–4]. However, the use of speckle-tracking echocardiography (STE) is not limited to the assessment of deformation of the LV myocardium and may be used to study myocardial motion in other chambers—including the assessment of left atrial (LA) and right ventricular (RV) strain. This review seeks to highlight the diagnostic, prognostic, and clinical significance of these parameters in various clinical conditions such as heart failure, atrial fibrillation, diastolic dysfunction, pulmonary hypertension, tricuspid regurgitation, and heart transplant recipients.

## Left Atrial Strain

### Physiology

**Fig. 1:**
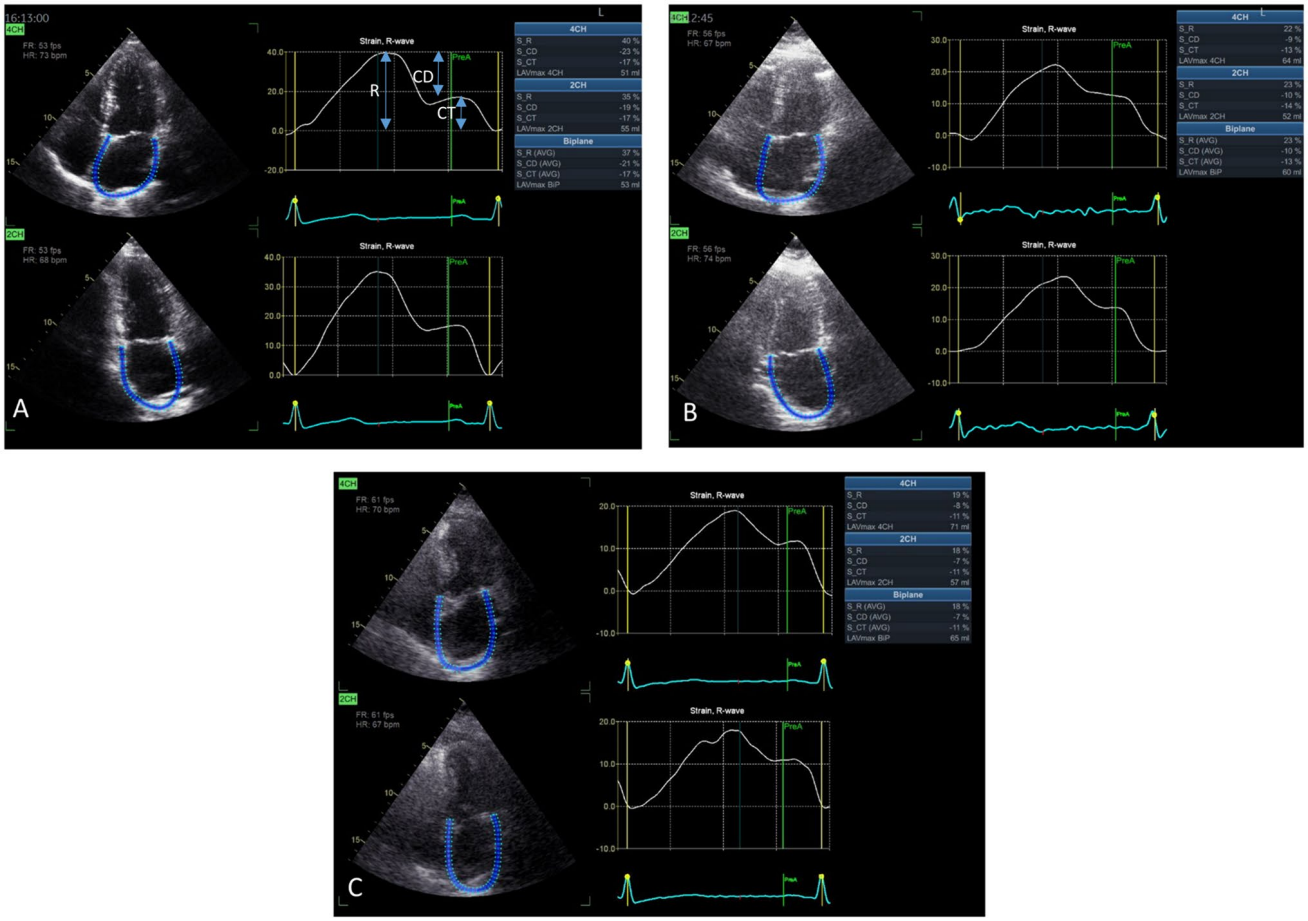
Examples of LA strain curves. **A** Healthy subject; LA volume (53 mls) is normal, as are reservoir (S_R), conduit (S_CD), and contractile strain (S_CT). **B** Heart failure; in the presence of indeterminate left atrial pressure (normal LA volume, but borderline E/e’, and no tricuspid regurgitation jet), LA reservoir strain > 18% is reassuring that LA pressure is normal. More reassuring still would have been an LA contractile strain > 14% [15••]. **C** Risk of atrial fibrillation. LA volume is borderline. The reservoir strain (18%) is associated with a > sixfold increased hazard of developing AF, compared with people with a reservoir strain > 23%. In absolute risk terms, this would be a rate of > 4% per year vs. < 1% per year [50]

Left atrial strain is a feasible and rapid speckle-tracking tool for assessing atrial function, which has been shown to have diagnostic and prognostic value. Each of the phases of atrial function can be measured with strain imaging, and there are normal ranges (Appendix Table 1) for reservoir function (which accommodates pulmonary venous return during ventricular systole), conduit function (which accommodates pulmonary venous return during early diastole), and contractile function (which facilitates ventricular filling during atrial systole) [5, 6] (Fig. 1). Nonetheless, it is important to appreciate that none of these parameters purely assess atrial function, because of the interplay between atrial and ventricular activity, preload, and afterload. Thus, reservoir function is determined by atrial compliance during ventricular systole, but is also governed by LV end-systolic volume and LV base descent during systole. Likewise, the LA and LV freely communicate during diastole, so conduit function is dependent on LV relaxation and stiffness (chamber compliance) and as well as on reservoir function and atrial compliance. The atrial booster pump (contractile strain) is mainly related to the effectiveness of atrial contractility but the quality of pump function depends on LV systolic reserve, atrial preload (degree of venous return), and atrial afterload (LV end-diastolic pressures) [5, 6].

### Techniques for Assessing LA Function

A number of imaging tools may be used for the assessment of LA size and function, but echocardiography remains the most accessible and least expensive [5]. While the assessment of LA size with either 2D or 3D measurement of LA volume indexed to body surface area (LAVI) is ubiquitous, volumetric analysis of the LA at multiple phases of the cardiac cycle is difficult. Measurement of LA volumes at their largest (at LV end-systole), smallest (at LV end-diastole), and immediately before atrial systole (at the onset of the P wave on the electrocardiogram) estimate the reservoir, conduit, and booster pump functions. Calculation of total, passive, and active emptying fractions, which pertain to the reservoir, conduit, and booster pump functions, is possible based on the given volumes [5, 6]. However, the volumetric approach is less feasible than STE for assessing LA function. Nonetheless, technical considerations are important for STE, including good image quality and an appropriate frame-rate (preferably 50–70 frames per second) (Fig. 1A). Potential technical challenges include limited endocardial resolution in the far field of apical views, thin atrial walls, and the existence of adjacent structures such as pulmonary veins or LA appendage.

Other imaging modalities for LA assessment include cardiac magnetic resonance (CMR) and cardiac computed tomography (CCT). In addition to LA anatomy and function, CMR is also able to assess LA fibrosis from late gadolinium enhancement (LGE) [7, 8]. CCT also permits the evaluation of pulmonary venous anatomy [9], estimation of risk of AF recurrence, and coronary artery disease [8].

### LA Strain in the Assessment of LV Diastolic Function

Echocardiography remains the primary imaging technique for the assessment of LV diastolic dysfunction (DD), by determination of LV relaxation and compliance, and the estimation of LV filling pressure [10]. LA strain is significantly and negatively associated with the severity of LVDD [11–13], and conversely, the severity of DD is linked to the prevalence of abnormal LA strain values [12, 13]. LA strain will become in important adjunct in the estimation of LV filling pressure. In patients with a mitral E/A ratio from 0.8 to 2.0, LV filling pressure is likely raised when two of the three predictive features (average E/e’ > 14, peak TR velocity > 2.8 m/s, and LAVi > 34 ml/m^2^) are present. If one of the three criteria is absent and the remaining two provide conflicting results, LA reservoir strain can serve as the third parameter. The effectiveness of this approach was demonstrated in a recent multicenter study, which utilized invasive LV filling pressure as a reference point [14, 15••]; LA reservoir strain < 18% corresponded to an increased LV filling pressure. However, LA strain is more difficult to use in the assessment of LV filling pressure in AF.

LA contractile strain may be an even stronger correlate of LV filling pressure than LA reservoir strain. The associations of both LA reservoir and booster strains with LV filling pressure are modest in patients with normal LV ejection fraction. In such circumstances, high values of LA booster strain accurately identify non-elevated LV filling pressure [14]. However, the disadvantage of using LA contractile strain as a marker of high LV filling pressure is that low values may also reflect atrial stunning post-atrial arrhythmias. Lower velocities of septal and lateral a’ (which show mitral annular velocity during LA contraction), and reduced transmitral A wave (determined by atrial contraction and LV compliance), may be confirmatory of impaired LA function.

LAVi is a reliable parameter for assessing the long-term effect of increased LV filling pressure on the LA. However, it has been demonstrated that LAVi combined with LA strain significantly improves the detection rate of LV diastolic dysfunction and increased LV filling pressure compared to using only LAVi. This finding is relevant for patients with preserved ejection fraction, among whom elevated LV filling pressure is indicated by reductions in LA reservoir strain and LA pump strain [16]. Moreover, LA reservoir strain has a stronger association with invasive LV filling pressure than does LAVi [17, 18]. Importantly, the measurement of LA reservoir strain using STE has shown very high feasibility, with a rate of ~ 95% [14].

### Prognostic Role of LA Strain in Heart Failure

Diastolic dysfunction and raised LV filling pressures are markers of adverse outcome in people with and without symptomatic heart failure. Left atrial strain delivers a practical and reliable diagnostic tool to identify elevated LA pressure both during rest and exercise in patients with HF or suspected HF (Fig. 1B). Moreover, LA strain demonstrates the highest prognostic value in patients with HF among the other non-invasive indices of filling pressure [19]. LA strain represents a robust approach for evaluating pulmonary artery wedge pressure (PAWP) at rest. Furthermore, it demonstrates consistent capability in detecting pathological increases in PAWP during exertion, even when resting pressures appear normal [19].

The progressive worsening of exercise capacity from stage A through stage B to stage C in HF is accompanied by a gradual impairment of LA reservoir and contractile strain and strain reserve. Individuals without HF with reduced resting LA reservoir strain and without the ability to increase LA reservoir strain after passive leg raise have been found to have a shorter 6-min walk distance than those with preserved atrial strain and strain reserve [20]. Peak VO_2_ is directly associated with LA compliance. LA structure and function are associated with non-specific HF symptoms in stages A and B, even after adjustment for comorbidities, risk factors, echocardiographic parameters of cardiac structure, and diastolic function.

In patients with heart failure with preserved ejection fraction (HFpEF), LA strain seems superior to LAVi for predicting adverse outcomes. Among patients with HFpEF, LA strain is more strongly associated with adverse outcomes than longitudinal LV and RV strain measurements [21]. Similarly, in patients with heart failure with reduced ejection fraction (HFrEF), reduced peak atrial longitudinal strain (PALS) was significantly associated with more advanced HF and greater impairment in both LV systolic and diastolic function indices. Impaired PALS strongly predicts adverse outcomes, independent of other clinical and echocardiographic factors used to assess prognosis. Moreover, PALS provides additional prognostic information concerning LAVi, LV filling pressures, and LV global longitudinal strain (GLS) [22].

While an increase in LAVi can indicate the chronic impact of elevated LV or LA filling pressures and be a predictor of adverse outcomes in patients with HFrEF [23], LA enlargement can also occur in patients with normal filling pressures, i.e., healthy athletes or individuals with lone arrhythmias. Therefore, measurements of LA dimensions may not always offer a dependable estimation of LA pressure or function [22].

### Detection of Atriopathy and Prediction of AF with LA Strain

Cardiac output can be reduced by around 15–20% by loss of atrial contraction when atrial fibrillation occurs in the context of HF. Conversely, increase of the LA booster pump function may compensate for decreased early filling in patients with impaired LV relaxation. In this setting, the likely reason for increased LA contractility is increased LA volume (Frank-Starling’s law). However, prolonged exposure to increased LV filling pressure leads to extreme dilatation and exhaustion of the Frank-Starling response, with both impaired LA function and the risk of AF [24]. A significant evidence base has been gathered regarding the prediction of AF with LA strain (Appendix Table 2).

In HFpEF, patients who develop AF are characterized by reduced LA strain (Fig. 1C)—independent of older age, higher BNP, and creatinine levels, increased LAVi, LV mass, impaired diastolic function, as well as reduced exercise capacity. Peak atrial contractile strain (PACS), peak atrial longitudinal strain (PALS), and LAVi demonstrate the highest predictive value for AF (PACS and PALS were independent of clinical data, LAVi, and E/e’ ratio). These three parameters (PACS, PALS, and LAVi) identify three key predictors of atrial fibrillation, and their combination is able to effectively distinguish high and low AF risk in HFpEF. This high-risk subset demonstrated a 33-fold increase in hazard [25].

The recurrence of AF after ablation is an important problem. In patients in sinus rhythm, LA reservoir and LA conduit strain have been strongly associated with atrial fibrillation recurrence, independent of LAVi, BMI, and LA pressure. However, some evidence points toward left atrial appendage velocity (LAAV), obtained at transesophageal echocardiography, as the strongest independent predictor [26]. In patients with AF, LAVi has been proposed as the only independently associated risk factor for AF recurrence after ablation, and LA strain appears to be less useful.

LA strain may help identify a group of patients with a high risk of developing AF. The appropriate clinical response to this predictor needs further study. It seems likely that pre-emptive lifestyle intervention, including stopping alcohol intake, may reduce the risk of progression. Whether there is sufficient justification to initiate empirical anticoagulation in this setting also requires further study [27].

## Right Ventricular Strain

### Right Ventricular (RV) Physiology

The right ventricular ejection fraction (or its surrogates including RV fractional area change) is a crucial factor in assessing the prognosis of a variety of conditions [28]. The muscle fibers within the RV are arranged in two layers—a superficial layer arranged circumferentially and a deeper layer arranged longitudinally. This specific arrangement determines the contractile motion of the RV, which is typically characterized by longitudinal shortening rather than the twisting and torsional contraction observed in the LV. In comparison to the LV, the RV is relatively thinner and more influenced by external factors, particularly pulmonary vascular resistance (PVR). Even slight increases in PVR can lead to significant reductions in RV cardiac output. However, in cases of chronic pressure overload, such as pulmonary hypertension (PH), the RV undergoes remodeling and hypertrophy as an adaptive response before eventually thinning and progressing toward failure [29].

### RV Strain vs. Other Parameters of RV Function

Multiple acoustic windows should be used to provide a precise evaluation of the RV. In routine clinical practice, RV strain appears more reliable than other, commonly used indices of RV longitudinal function, including tricuspid annular plane systolic excursion (TAPSE) and annular systolic velocity (s’) from Doppler tissue imaging (DTI). The principal problem with standard parameters of longitudinal function is that they may be influenced by tethering—i.e., passive motion of the RV by preserved LV function. In certain conditions such as regional RV dysfunction, severe pulmonary arterial hypertension, or following cardiac surgery, TAPSE may not provide accurate measurements. Despite its limitations, TAPSE remains widely utilized as an index for assessing RV performance due to its ease of measurement, reproducibility, and diagnostic and prognostic significance across various disease states [30]. Similar to TAPSE, s’ reflects the function of longitudinal fibers, which play a significant role in RV contraction. The advantages and limitations of s’ are comparable to those observed with TAPSE; however, s’ demonstrates a stronger correlation with RV ejection fraction measured by cardiac magnetic resonance imaging (CMR) than does TAPSE [31]. RV longitudinal and radial function are often not matched, so it is still helpful to assess fractional area change (FAC). FAC offers insights into both the longitudinal and radial aspects of RV contraction. Unlike other methods, it is not limited to a single type of motion. However, one significant limitation of this technique is the often poor visualization of the RV lateral wall.

**Fig. 2:**
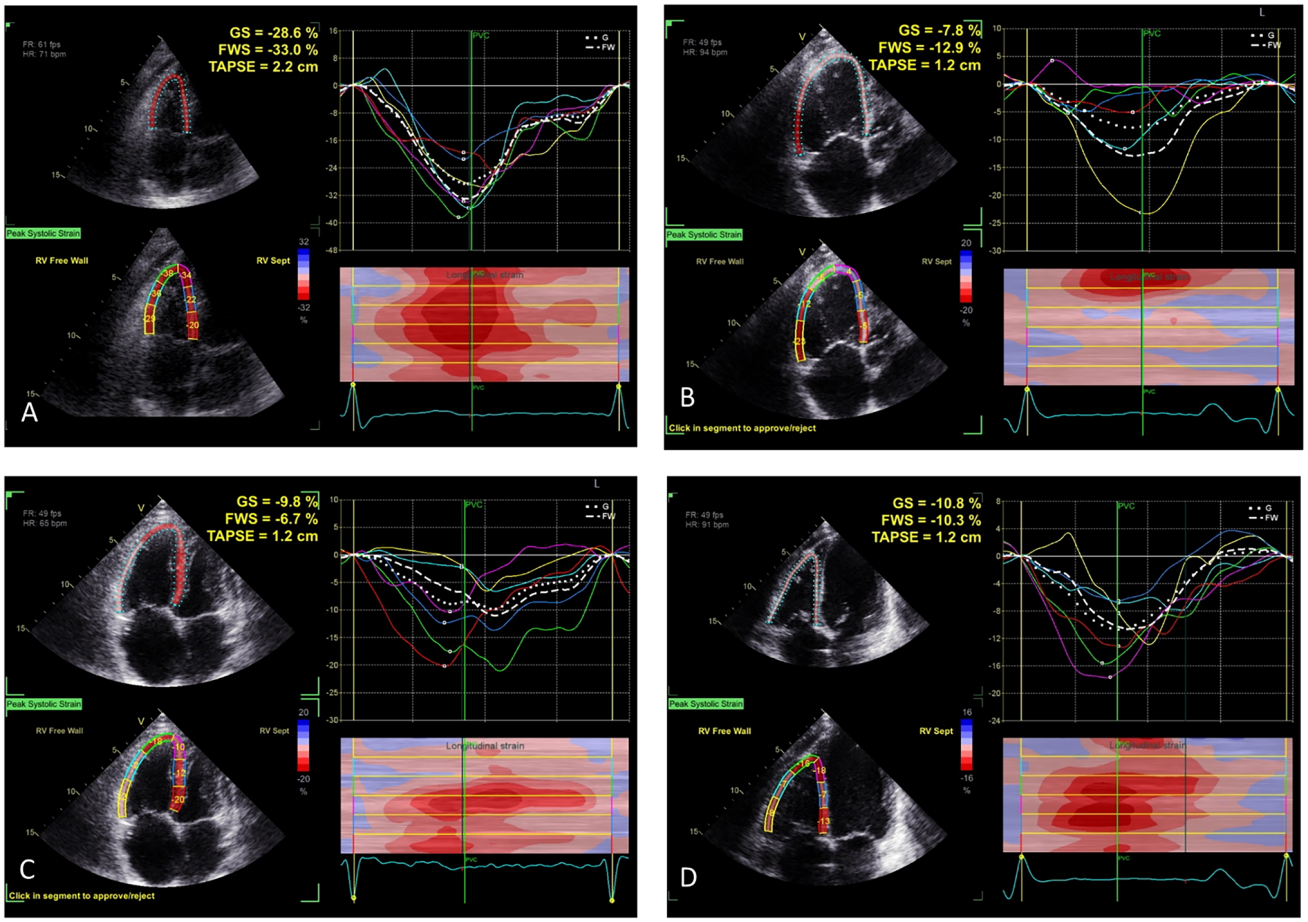
Examples of RV strain curves. **A** Healthy subject; global strain (GS, lower limit of normal [LLN] − 17%) and free-wall strain (FWS, LLN − 19%) are normal [37••]. **B** Heart failure. **C** Pulmonary hypertension. **D** Tricuspid regurgitation. These patients with RV enlargement have both impaired GS and FWS, and this is prognostically meaningful

In comparison to the abovementioned parameters, RV strain (percent change in myocardial length) reflects both global and regional systolic function (Fig. 2A), but is significantly affected by image quality. RV strain is a strong independent predictor of severe adverse events in chronic heart failure and probably outperforms other indices of RV systolic function [32].

### Right Ventricular Strain in the Assessment of RV/PA Coupling

The function of the RV, which involves adapting to increased afterload, plays a critical role in determining the progression and symptoms of all types of pulmonary hypertension (PH). Nevertheless, as afterload continues to rise, the initially adaptive response transforms into pathological maladaptive remodeling. The current gold-standard approach for evaluating RV-PA coupling involves invasive and technically complex procedures. There is a need for non-invasive techniques, primarily relying on echocardiography, to estimate RV-PA coupling [33]. The ratio of TAPSE and estimated pulmonary arterial systolic pressure (TAPSE/PASP) was among the earliest and most well-validated non-invasive Doppler echocardiography-derived substitutes for RV-PA coupling. Currently TAPSE is being replaced by diverse echocardiographic parameters of RV contractility, especially RV strain. Initially employed as a prognostic test in heart failure [34•], it has also been used to assess prognosis in precapillary PH [35]. It is also a predictor of critical endpoints in tricuspid regurgitation.

### Right Ventricular Strain in Heart Failure (Appendix Table 3)

Approximately 20% of patients with **HFpEF** have RV dysfunction, and this prevalence may be as high as 30–50% [36]. There is a progressive decline in RV free wall longitudinal strain (RVFWLS) across different groups with varying degrees of LV diastolic dysfunction (Fig. 2B). RVGLS has a stronger correlation with CMR-derived RVEF than do other conventional echocardiographic parameters, making it a more accurate measure of RV function. RVGLS was more impaired in HFpEF patients when compared to asymptomatic patients with LV diastolic dysfunction. Furthermore, reduced RVGLS was significantly associated with worse NYHA functional class in HFpEF patients, and it is an independent predictor of both overall mortality and heart failure rehospitalization.

In **HFrEF**, RVGLS and RVFWLS have been independently associated with death, cardiovascular death, heart transplantation, and worsening HF. Impaired RVGLS has been linked to worsening NYHA class, increased LV volume, and worse LV diastolic function. Interestingly, RVFWLS demonstrates superior incremental value over RVGLS in HFrEF patients, perhaps because the septal contribution to RVGLS is already assessed in LV parameters. Both RVGLS and RVFWLS can be reduced, even if traditional parameters like TAPSE and s’ are within the normal range. Strain levels are related to the clinical status of stable HF patients, provide information about global RV systolic function, and enable the detection of subclinical RV dysfunction [37••].

In patients with **acute HFrEF**, RVFWLS is correlated with conventional RV function parameters (TAPSE, s’, RVFAC) [38]. In this group of patients, RVFWLS is an independent predictor of poor prognosis, offering incremental prognostic value beyond clinical, and standard echocardiographic parameters.

### Right Ventricular Strain in Pulmonary Hypertension (Appendix Table 4)

Similar to HF patients, in pulmonary arterial hypertension (PAH), conventional RV systolic function parameters like TAPSE, FAC, s’, and RVEF may appear normal despite abnormal RV strain. This highlights the importance of using RV strain as it can detect subclinical RV dysfunction in the early stages of the disease [39]. RVFWLS (Fig. 2C) more accurately predicts cardiovascular events in PAH than do TAPSE, FAC, s’, and RV index of myocardial performance and is an independent predictor of adverse clinical events and mortality. Individuals with a significantly impaired RVFWLS showed a proportional worsening in functional class, shorter 6-min walk distance, and higher N-terminal pro-B-type natriuretic peptide level. Individuals who experience an improvement in RVFWLS in response to therapy have a 7 times lower mortality rate, confirming the better prognosis in patients with PAH if RV function improves during therapy [40]. Conversely, a relative reduction of RV longitudinal strain > 10% was identified as a significant and independent risk factor for adverse outcomes in patients with PH.

### Right Ventricular Strain Assessment in Tricuspid Regurgitation (Appendix Table 5)

The reduction in RVFWLS is directly related to the severity of tricuspid regurgitation [41]. Decreased RVFWLS (Fig. 2D), TAPSE, and FAC have been identified as independent predictors of 2-year all-cause mortality, with RV strain showing the highest predictive value [42, 43]. RVFWLS is independently associated with 2.8-year all-cause mortality and provides additional prognostic information beyond FAC and TAPSE. Moreover, RVFWLS remains independently associated with outcome after adjusting for comorbidities (diabetes mellitus, chronic kidney disease, coronary artery disease) and New York Heart Association class III/IV, whereas FAC and TAPSE did not show independent predictive value. Patients with impaired CMR-derived RVFWLS exhibited significantly lower survival rates than those with preserved RVFWLS—even after accounting for clinical and imaging risk factors, including RV size and ejection fraction [44].

While data on RV strain in predicting survival after valvular “edge-to-edge” repair procedures is still being investigated, it holds promise as a useful parameter in patients with tricuspid regurgitation, and the body of evidence supporting its relevance continues to grow.

### Ventricular Strain in Heart Transplant Recipients

After heart transplantation, RV failure stands as a crucial factor contributing to early cardiac morbidity and mortality. As previously mentioned, myocardial strain is sensitive to the detection of subclinical impairment of RV function, even when other conventional RV function parameters appear normal. In a study investigating heart transplant recipients using CMR-derived RVLS and RVEF, RVLS was decreased in recipients with adverse events compared to those without such events. Moreover, both RVEF and RVLS emerged as significant independent predictors in heart transplant recipients. Notably, the predictive value of RVLS was superior to that of RVEF [45]. In a separate study, it was demonstrated through multivariable and univariable analyses that RVGLS serves as a significant predictor of 6-year all-cause mortality. Additionally, the RV diastolic strain rate (the rate of change of strain over time, which is reduced when the rate of contraction is reduced) was found to be a reliable predictor for both all-cause mortality and post-heart transplantation complications. Interestingly, while novel strain parameters of myocardial function showed predictive value, traditional 2D and Doppler parameters of systolic and diastolic function did not exhibit significant differences between rejection score groups [46]. These findings suggest that strain assessment in patients after heart transplantation could serve as a valuable, noninvasive tool for identifying individuals with a poor prognosis. This approach has the potential to enable early optimization of treatment strategies.

In pediatric pulmonary hypertension, 3D echocardiographic RVEF, RVFWLS, and FAC have been demonstrated as predictors of outcomes [47]. Moreover, a recent meta-analysis revealed that RVGLS, assessed using both 3D and 2D echocardiography, was a survival predictor in patients with pulmonary hypertension [48]. Additionally, the inclusion of RV dyssynchrony (measured based on 2D echocardiographic longitudinal strain) in a multivariable model improved the prediction of peak oxygen uptake, even though the model already incorporated conventional parameters such as RV FAC [49]. These findings highlight the significance of RV strain in pulmonary hypertension management.

## Conclusions

In individuals with and without HF, impaired left atrial function, absence of LA functional reserve, and worse LV diastolic function are linked to decreased submaximal exercise capacity. Initiating treatments targeting these functional aspects may enhance exercise capacity and potentially prevent the onset of heart failure. Early identification of patients with a high risk of AF may lead to preventive strategies and possibly even empirical anticoagulation.

RV function has significant clinical and prognostic implications across various clinical scenarios, including HF, PH, tricuspid regurgitation, or in heart transplant recipients. The introduction of RV (global and free wall) longitudinal strain as a novel echocardiographic parameter has enhanced the assessment of RV systolic function, enabling the detection of subclinical RV dysfunction. However, additional studies are necessary to fully establish its role in patient management.

### Supplementary Information

Below is the link to the electronic supplementary material.

Supplementary file1 (DOCX 100 KB)

## Data Availability

No datasets were generated or analyzed during the current study.
