# Supplementary material for: Evolving Applications of Echocardiography in the Evaluation of Left Atrial and Right Ventricular Strain
Source: Curr Cardiol Rep. 2024 Apr 22;26(6):593–600. doi: 10.1007/s11886-024-02058-x (PMC11199230; doi:10.1007/s11886-024-02058-x)
Supplement: Supplementary file 1 — Supplementary file1 (DOCX 100 KB) [file 11886_2024_2058_MOESM1_ESM.docx]

| **Appendix Table 1: Normal Values for LA Strain** | | | | | | |
| --- | --- | --- | --- | --- | --- | --- |
| **First Author**  **(Ref. #)** | **Design** | **N** | **Population** | **Outcome** | **Analysis Software** |  |
| Morris et al (2015) (1) | Prospective, observational | 329 | Healthy controls | Normal LA strain was 45.5+11.4% | EchoPAC, GE |  |
| Sugimoto et al (2018)(2) | Prospective, observational | 371 | Healthy controls | LA reservoir strain was 42.5 (36.1-48.0%)  LA conduit function was 25.7% (20.4-31.8%)  LA contractile strain was 16.3% (12.9-19.5%) | TomTec Imaging System |  |
| D’Ascenzi et al (2019) (3) | Meta-analysis | 2,087 | Healthy controls | LA reservoir strain was 38 +/-3% (95% CI, 32-43%) | Various |  |
| Sun et al (2020) (4) | Prospective, observational | 324 | Healthy controls | LA reservoir strain was 35.9 +/- 10.6%  LA conduit strain was 21.9 +/- 9.3%  LA contractile strain was 13.9+/-3.6% | EchoPAC and BT 201 device, GE |  |
| Nielsen et al (2021) (5) | Prospective, observational | 1641 | Healthy controls | LA reservoir strain was 39.4% (23.0-67.6%)  LA conduit strain was 23.7% (8.8-44.8%)  LA contractile strain was 15.5% (6.4-28.0%) | EchoPAC, GE |  |
| Pathan et al (2017) (6) | Meta-analysis | 2542 | Healthy patients | LA reservoir strain was 39% (95% CI, 38-41)  LA conduit strain was 23% (95% CI, 21-25%)  LA contractile strain was 17% (95% CI, 16-19%) | Various |  |
| Genovese et al (2023) (7) | Prospective | 25 | Healthy patients | LA reservoir, conduit, and contractile strain values decreased with reduction in preload (reservoir: 42.9 to 27.5 (p < .001); conduit: 29.3 to 20.2 (p < .001); contractile: 13.6 to 7.3 (p < .001). | TomTec |  |

| **Appendix Table 2: Use of LA Strain to Predict AF** | | | | | | | | |
| --- | --- | --- | --- | --- | --- | --- | --- | --- |
| **First Author**  **(Ref. #)** | **Design** | **N** | **Population** | **Outcome** | **Strain Cutoff (%)** | **Size of effect or Test Performance** | **Analysis Software** |  |
| Hauser et al. (2021)(8) | Prospective | 4466 | Healthy individuals | Incident AF | Left atrial reservoir strain <32%, lowest tertile vs >41.1%, highest tertile. | HR 1.05, 95% confidence interval (CI) (1.03-1.07), P < 0.001, per 1% decrease] | Vivid 9, GE |  |
| Park et al. (2020) (9) | Prospective | 4312 | Patients with acute heart failure | Incident AF | Left atrial reservoir strain was <18% | HR: 1.60; 95% CI: 1.18 to 2.17 | TomTec Imaging Systems |  |
| Raman et al (2021) (10) | Prospective | 238 | Patients with hypertrophic cardiomyopathy | Incident AF | Left atrial reservoir strain was ≤ 18% | HR 2.56, 95% CI 1.24-5.27 | *CMR* |  |
| Ramkumar et al (2021) (11) | Prospective | 351 | Patients ≥65 years with ≥1 risk factor for AF | Incident AF | Left atrial reservoir strain was 28 ± 11% vs. 35 ± 8% | p < 0.001 | ImageArena, Tomtec Imaging systems |  |
| Jasic-Szpak et al (2021) (12) | Prospective | 170 | Patients with HFpEF | Incident AF | Classification and regression trees analysis identified PACS ≤12.7%, PALS ≤29.4%, and LAVI >34.3 ml/m^2^ as discriminatory nodes for AF, with a 33-fold greater hazard of AF. | p < 0.001 | Vivid E9, GE |  |
| Mouselimis el al (2020) (13) | Meta-analysis | 880 | Paroxysmal and persistent AF | AF recurrence after catheter ablation | LAS_AFR_: 17.5 ± 8.7% vs. LAS_nAFR_: 24.1 ± 9.5%. A pooled cutoff value of 21.9% for LAS was extracted for the prediction of ablation success. | p < 0.00001 | Various |  |
| Khan et al (2023) (14) | Prospective | 83 | Pre ablation vs. Post ablation | AF recurrence comparison | LA contractile strain at 3 months was the only independent predictor of AF recurrence.  (9.2±3.4% versus 5.6±2.5%; *P*<0.001) | P<0.001 | Philips IE33 and GE Vivid E9/E95 machines |  |
| **AF in Embolic Stroke of Unknown Source** | | | | | | | | |
| **First Author**  **(Ref. #)** | **Design** | **N** | **Population** | **Outcome** | **Strain Cutoff (%)** | **Size of effect or Test Performance** | **Analysis Software** |  |
| Rasmussen et al (2019) (15) | Retrospective, cohort | 186 | Cryptogenic stroke patients | Episode of AF following stroke | Left atrial reservoir strain was 27% (vs control 35%) | OR 1.13 [1.04; 1.22], p = 0.003 | Echopac, GE Medical |  |
| Deferm et al (2021) (16) | Retrospective | 191 | Cryptogenic stroke patients | Episode of AF following stroke | Left atrial contractile strain was strongest predictor of AF | OR 2.88 per SD increase 95% confidence interval, 1.29-6.41; P = .010 | ImageArena, Tomtec Imaging systems |  |
| Ble et al (2021) (17) | Prospective | 75 | Cryptogenic stroke patients | Episode of AF following stroke | Left atrial reservoir strain was 19.6 ± 5.7% (vs control vs. 29.5 ± 7.2%)  Left atrial contractile strain was 8.9 ± 3.9% vs. control 16.5 ± 6% | *p* < 0.001 | Vivid E9 GE |  |
| Ramkumar et al (2021) (11) | Prospective | 453 | Cryptogenic stroke patients | Episode of AF following stroke | Left atrial reservoir strain was predictivearea under ROC curve 0.83 vs 0.57 | p < 0.001 | ImageArena, Tomtec Imaging systems |  |
| Kawakami et al (2020) (18) | Prospective | 531 | Cryptogenic stroke patients | Episode of AF following stroke | Left atrial reservoir strain was predictive, area under the curve was 0.851  Left atrial contractile strain area under the curve was 0.825 | p < 0.01 | TomTec Imaging Systems |  |
| **Prediction of AF Post Cardioversion** | | | | | | | | |
| **First Author**  **(Ref. #)** | **Design** | **N** | **Population** | **Outcome** | **Strain Cutoff (%)** | **Size of effect or Test Performance** | **Analysis Software** |  |
| Moreno-Ruiz et al (2019) (19) | Prospective | 131 | Patients post cardioversion | Episode of AF following cardioversion | Left atrial reservoir strain ≤ 10.75% | HR 8.89 [(2.2–35.7), p < 0.01 | Philips iE33 |  |
| Walek et al (2020) (20) | Prospective | 89 | Patients post cardioversion | Episode of AF following cardioversion | Left atrial contractile strain was predictive.  Odds ratio (OR) 0.78; 95%CI 0.63-0.97; | P = .027 | Vivid S6, GE |  |
| **Prediction of AF Post Ablation** | | | | | | | | |
| **First Author**  **(Ref. #)** | **Design** | **N** | **Population** | **Outcome** | **Strain Cutoff (%)** | **Size of effect or Test Performance** | **Analysis Software** |  |
| Motoc et al (2021) (21) | Prospective | 172 | Post Cryoablation | Episode of AF post ablation | Left atrial reservoir strain ≤ 17% | HR = 9.45, 95%CI: 3.17-28.13, p < 0.001 | Vivid E95, GE |  |
| Nielsen et al (2020) (22) | Meta-analysis | 1025 | Post Radiofrequency Ablation | Episode of AF post ablation | Left atrial reservoir strain was a significant predictor of AF recurrence  OR: 1.16, CI95% [1.09–1.24], | p < 0.001, per 1% decrease | Various |  |
| Ma et al (2016) (23) | Meta-analysis | 686 | Post Ablation | Episode of AF post ablation | Left atrial strain mean was 18.4% [8.8-24.5%] versus 25.3% [13.6-32.7%] | P < 0.001 | Various |  |
| Mouselimis et al (2020) | Meta-analysis | 880 | Post Ablation | Episode of AF post ablation | Left atrial strain was 17.5 ± 8.7% vs. control 24.1 ± 9.5%, | p < 0.00001 | GE and TomTec |  |
| Soysal et al (2023) (24) | Prospective | 77 | Pre ablation vs. Post ablation | Mechanical function of LA comparison | LA reservoir strain and LA contractile strain showed significant recovery after the procedure.  (28.3 ± 12.8 vs. 34.6 ± 13.8, and −10.8 ± 7.9 vs. −13.9 ± 9.3 respectively) | (*p* < .001 and *p* = .014 respectively) | Qlab, Philips |  |
| Khan et al (2023) (14) | Prospective | 83 | Pre ablation vs. Post ablation | Mechanical function of LA comparison | Higher reservoir strain (22.6±8.5% versus  16.7±5.7%; ), and contractile strain (9.2±3.4% versus 5.6±2.5%; ) were  noted in the sinus rhythm compared with AF recurrence group following ablation at 3  months. | P=0.001  P<0.001 | Philips IE33 and GE Vivid E9/E95 machines |  |

| **Appendix Table 3.** Predictive value of RV longitudinal strain in patients with heart failure | | | | | |
| --- | --- | --- | --- | --- | --- |
| **First Author**  **(Ref. #)** | **N** | **Population** | **RV stain cut-off** | **Outcome** |  |
| Morris et al. (25) | 201 | Patients with diastolic dysfunction or/and HFpEF | −14.41% ±3.80 | 75% of HfpEF patients had impaired RV GLS which was significantly associated with worse NYHA class. HfpEF patients had lower RVGLS than asymptomatic patients with LV diastolic dysfunction. |  |
| Lejeune et al. (26) | 149 | Patients with HFpEF | >-17,5% | RVGLS provided significant prognostic value; TAPSE and FAC did not. RVGLS showed great correlation with RVEF by CMR. Mean RVGLS was significantly worse in patients with HFpEF compared with control subjects. |  |
| Iacoviello et al. (27) | 332 | Patients with HFrEF | RVGLS: −14.6 ± 4.6 RVFWLS: −21.5 ± 6.2 | Both RV GLS and RVFWLS independently predicted all-cause mortality. |  |
| Motoki et al. (28) | 171 | Patients with HFrEF | >-14.8% | After adjustment for age, LVEF, RV s', E/e' septal, and right atrial volume index RV strain ≥ -14.8% predicted adverse events. Worse RV strain was associated with increasing NYHA class and greater LV volume, reduced LVEF, worse LV diastolic dysfunction, left atrial volume index, and standard indices of RV systolic and diastolic dysfunction, right atrial volume index. |  |
| Morris et al. (29) | 880 | Patients with HFrEF, HFpEF, asymptomatic patients, and healthy controls | RVGLS -24.5% ± 3.8 RVFWLS -28.5% ± 4.8 | Both RV GLS and RVFWLS were significantly linked to the symptomatic status of the patients. The normal range of RV systolic strain in the healthy subjects was 24.5% ± 3.8 for RV global strain -and -28.5% ± 4.8 for RVFWLS (lowest expected value -17 and -19%, respectively). |  |
| Borovac et al. (30) | 42 | Patients with acute HF | RVFWLS: -16.5% | Patients with impaired RVFLS had worse hepatic insufficiency parameters. Worse RVFWLS is independently associated with a higher degree of hepatic dysfunction.  Additionally, RVFWLS had a positive correlation with other conventional RV function parameters like TAPSE, FAC, s’. |  |
| Hamada-Harimura et al. (31) | 618 | Patients with acute decompensated HF | RVFWLS: ≥-13.1% | Impaired RVFWLS was independently associated with cardiac events even after adding RVFWLS to clinical risk model (age, NYHA III/IV, BUN, BNP). |  |

HFpEF – Heart Failure with preserved ejection fraction; RV – right ventricle; GLS – global longitudinal strain; NYHA – New York Heart Association; LV – left ventricle; TAPSE - tricuspid annular plane systolic excursion; FAC – fractional area change; EF – ejection fraction; CMR – cardiac magnetic resonance; RVFWLS – right wall free wall longitudinal strain; ALP - Alkaline phosphatase; ALT - alanine transaminase; BUN - blood urea nitrogen; BNP – brain natriuretic peptide

| **Appendix Table 4.** Predictive value of RV longitudinal strain in patients with pulmonary hypertension | | | | |
| --- | --- | --- | --- | --- |
| **First Author**  **(Ref. #)** | **N** | **Population** | **RV stain cut-off** | **Outcome** |
| Motoji et al. (32) | 42 | Subjects with pulmonary hypertension | -19.4% | RVFWLS was better predictor of CV events than TAPSE, FAC, s’, and RV index of myocardial work in terms of specificity and sensitivity. |
| Park et al. (33) | 51 | Patients with pulmonary arterial hypertension without atrial fibrillation | -15.5% | RV GLS was an independent predictor of mortality and adverse clinical events. |
| Fine et al. (34) | 575 | Patients with known or suspected pulmonary hypertension | -15% | Prediction of survival by RVFWLS after adjustment for pulmonary pressure, right atrial pressure, and pulmonary vascular resistance. |
| Haeck et al. (35) | 150 | Subjects with pulmonary hypertension of different etiologies | -19% | RVFWLS ≥-19% independently predicted all-cause mortality, distinguishing it from TAPSE and FAC. |
| Hardegree et al. (36) | 50 | Subjects with pulmonary hypertension | -12.5% | ≥5% improvement in RVFWLS yield >7-fold lower mortality. |
| Hulshof et al. (37) | 1169 | Subjects with pulmonary hypertension | 10% | Relative reduction of RVLS >10% was identified as a significant and independent risk factor for adverse outcomes in patients with PH. |

RVFWLS – right wall free wall longitudinal strain; TAPSE - tricuspid annular plane systolic excursion; FAC – fractional area change; RV – right ventricle; GLS – global longitudinal strain; PH – pulmonary hypertension

| **Appendix Table 5.** Predictive value of RV longitudinal strain in patients with valvular heart disease | | | | |
| --- | --- | --- | --- | --- |
| **First Author**  **(Ref. #)** | **N** | **Population** | **RV strain cut-off** | **Outcome** |
| Bannehr et al. (38) | 1,089 | General population | -18% | Progressive increase from FAC, through TAPSE to RVFWLS in the prediction of all-cause mortality in terms of sensitivity and specificity. |
| Prihadi et al. (39) | 896 | Subjects with significant functional tricuspid regurgitation | −23% | RVSWLS was independently associated with all-cause mortality and was stronger than FAC and TAPSE. |
| Romano et al. (40) | 72 | Patients with severe functional tricuspid regurgitation | -16% | CMR RVGLS was an independent predictor of mortality after adjusting for comorbidities and imagining risk factors. |

FAC – fractional area change; TAPSE - tricuspid annular plane systolic excursion; RVFWLS – right wall free wall longitudinal strain; CMR – cardiac magnetic resonance; RV – right ventricle; GLS – global longitudinal strain;

1. Morris DA, Takeuchi M, Krisper M, Kohncke C, Bekfani T, Carstensen T, et al. Normal values and clinical relevance of left atrial myocardial function analysed by speckle-tracking echocardiography: multicentre study. Eur Heart J Cardiovasc Imaging. 2015;16(4):364-72.

2. Sugimoto T, Robinet S, Dulgheru R, Bernard A, Ilardi F, Contu L, et al. Echocardiographic reference ranges for normal left atrial function parameters: results from the EACVI NORRE study. Eur Heart J Cardiovasc Imaging. 2018;19(6):630-8.

3. D'Ascenzi F, Piu P, Capone V, Sciaccaluga C, Solari M, Mondillo S, et al. Reference values of left atrial size and function according to age: should we redefine the normal upper limits? Int J Cardiovasc Imaging. 2019;35(1):41-8.

4. Sun BJ, Park JH, Lee M, Choi JO, Lee JH, Shin MS, et al. Normal Reference Values for Left Atrial Strain and Its Determinants from a Large Korean Multicenter Registry. J Cardiovasc Imaging. 2020;28(3):186-98.

5. Nielsen AB, Skaarup KG, Hauser R, Johansen ND, Lassen MCH, Jensen GB, et al. Normal values and reference ranges for left atrial strain by speckle-tracking echocardiography: the Copenhagen City Heart Study. Eur Heart J Cardiovasc Imaging. 2021;23(1):42-51.

6. Pathan F, D'Elia N, Nolan MT, Marwick TH, Negishi K. Normal Ranges of Left Atrial Strain by Speckle-Tracking Echocardiography: A Systematic Review and Meta-Analysis. J Am Soc Echocardiogr. 2017;30(1):59-70 e8.

7. Genovese D, Singh A, Volpato V, Kruse E, Weinert L, Yamat M, et al. Load Dependency of Left Atrial Strain in Normal Subjects. J Am Soc Echocardiogr. 2018;31(11):1221-8.

8. Hauser R, Nielsen AB, Skaarup KG, Lassen MCH, Duus LS, Johansen ND, et al. Left atrial strain predicts incident atrial fibrillation in the general population: the Copenhagen City Heart Study. European Heart Journal - Cardiovascular Imaging. 2021;23(1):52-60.

9. Park JJ, Park JH, Hwang IC, Park JB, Cho GY, Marwick TH. Left Atrial Strain as a Predictor of New-Onset Atrial Fibrillation in Patients With Heart Failure. JACC Cardiovasc Imaging. 2020;13(10):2071-81.

10. Raman B, Smillie RW, Mahmod M, Chan K, Ariga R, Nikolaidou C, et al. Incremental value of left atrial booster and reservoir strain in predicting atrial fibrillation in patients with hypertrophic cardiomyopathy: a cardiovascular magnetic resonance study. J Cardiovasc Magn Reson. 2021;23(1):109.

11. Ramkumar S, Pathan F, Kawakami H, Ochi A, Yang H, Potter EL, et al. Impact of disease stage on the performance of strain markers in the prediction of atrial fibrillation. Int J Cardiol. 2021;324:233-41.

12. Jasic-Szpak E, Marwick TH, Donal E, Przewlocka-Kosmala M, Huynh Q, Gozdzik A, et al. Prediction of AF in Heart Failure With Preserved Ejection Fraction: Incremental Value of Left Atrial Strain. JACC Cardiovasc Imaging. 2021;14(1):131-44.

13. Mouselimis D, Tsarouchas AS, Pagourelias ED, Bakogiannis C, Theofilogiannakos EK, Loutradis C, et al. Left atrial strain, intervendor variability, and atrial fibrillation recurrence after catheter ablation: A systematic review and meta-analysis. Hellenic J Cardiol. 2020;61(3):154-64.

14. Khan HR, Yakupoglu HY, Kralj-Hans I, Haldar S, Bahrami T, Clague J, et al. Left Atrial Function Predicts Atrial Arrhythmia Recurrence Following Ablation of Long-Standing Persistent Atrial Fibrillation. Circ Cardiovasc Imaging. 2023;16(6):e015352.

15. Rasmussen SMA, Olsen FJ, Jørgensen PG, Fritz-Hansen T, Jespersen T, Gislason G, et al. Utility of left atrial strain for predicting atrial fibrillation following ischemic stroke. Int J Cardiovasc Imaging. 2019;35(9):1605-13.

16. Deferm S, Bertrand PB, Churchill TW, Sharma R, Vandervoort PM, Schwamm LH, et al. Left Atrial Mechanics Assessed Early during Hospitalization for Cryptogenic Stroke Are Associated with Occult Atrial Fibrillation: A Speckle-Tracking Strain Echocardiography Study. J Am Soc Echocardiogr. 2021;34(2):156-65.

17. Ble M, Benito B, Cuadrado-Godia E, Pérez-Fernández S, Gómez M, Mas-Stachurska A, et al. Left Atrium Assessment by Speckle Tracking Echocardiography in Cryptogenic Stroke: Seeking Silent Atrial Fibrillation. J Clin Med. 2021;10(16).

18. Kawakami H, Ramkumar S, Pathan F, Wright L, Marwick TH. Use of echocardiography to stratify the risk of atrial fibrillation: comparison of left atrial and ventricular strain. Eur Heart J Cardiovasc Imaging. 2020;21(4):399-407.

19. Moreno-Ruiz LA, Madrid-Miller A, Martínez-Flores JE, González-Hermosillo JA, Arenas-Fonseca J, Zamorano-Velázquez N, et al. Left atrial longitudinal strain by speckle tracking as independent predictor of recurrence after electrical cardioversion in persistent and long standing persistent non-valvular atrial fibrillation. Int J Cardiovasc Imaging. 2019;35(9):1587-96.

20. Wałek P, Ciesla E, Gorczyca I, Wożakowska-Kapłon B. Left atrial wall dyskinesia assessed during contractile phase as a predictor of atrial fibrillation recurrence after electrical cardioversion performed due to persistent atrial fibrillation. Medicine (Baltimore). 2020;99(49):e23333.

21. Motoc A, Luchian ML, Scheirlynck E, Roosens B, Chameleva H, Gevers M, et al. Incremental value of left atrial strain to predict atrial fibrillation recurrence after cryoballoon ablation. PLoS One. 2021;16(11):e0259999.

22. Nielsen AB, Skaarup KG, Lassen MCH, Djernæs K, Hansen ML, Svendsen JH, et al. Usefulness of left atrial speckle tracking echocardiography in predicting recurrence of atrial fibrillation after radiofrequency ablation: a systematic review and meta-analysis. The International Journal of Cardiovascular Imaging. 2020;36(7):1293-309.

23. Ma XX, Boldt LH, Zhang YL, Zhu MR, Hu B, Parwani A, et al. Clinical Relevance of Left Atrial Strain to Predict Recurrence of Atrial Fibrillation after Catheter Ablation: A Meta-Analysis. Echocardiography. 2016;33(5):724-33.

24. Soysal AU, Ozturk S, Onder SE, Atici A, Tokdil H, Raimoglu U, et al. Left atrial functions in the early period after cryoballoon ablation for paroxysmal atrial fibrillation. Pacing Clin Electrophysiol. 2023;46(8):861-7.

25. Morris DA, Gailani M, Vaz Pérez A, Blaschke F, Dietz R, Haverkamp W, et al. Right ventricular myocardial systolic and diastolic dysfunction in heart failure with normal left ventricular ejection fraction. J Am Soc Echocardiogr. 2011;24(8):886-97.

26. Lejeune S, Roy C, Ciocea V, Slimani A, de Meester C, Amzulescu M, et al. Right Ventricular Global Longitudinal Strain and Outcomes in Heart Failure with Preserved Ejection Fraction. J Am Soc Echocardiogr. 2020;33(8):973-84.e2.

27. Iacoviello M, Citarelli G, Antoncecchi V, Romito R, Monitillo F, Leone M, et al. Right Ventricular Longitudinal Strain Measures Independently Predict Chronic Heart Failure Mortality. Echocardiography. 2016;33(7):992-1000.

28. Motoki H, Borowski AG, Shrestha K, Hu B, Kusunose K, Troughton RW, et al. Right ventricular global longitudinal strain provides prognostic value incremental to left ventricular ejection fraction in patients with heart failure. J Am Soc Echocardiogr. 2014;27(7):726-32.

29. Morris DA, Krisper M, Nakatani S, Köhncke C, Otsuji Y, Belyavskiy E, et al. Normal range and usefulness of right ventricular systolic strain to detect subtle right ventricular systolic abnormalities in patients with heart failure: a multicentre study. Eur Heart J Cardiovasc Imaging. 2017;18(2):212-23.

30. Borovac JA, Glavas D, Susilovic Grabovac Z, Supe Domic D, Stanisic L, D'Amario D, et al. Right Ventricular Free Wall Strain and Congestive Hepatopathy in Patients with Acute Worsening of Chronic Heart Failure: A CATSTAT-HF Echo Substudy. J Clin Med. 2020;9(5).

31. Hamada-Harimura Y, Seo Y, Ishizu T, Nishi I, Machino-Ohtsuka T, Yamamoto M, et al. Incremental Prognostic Value of Right Ventricular Strain in Patients With Acute Decompensated Heart Failure. Circ Cardiovasc Imaging. 2018;11(10):e007249.

32. Motoji Y, Tanaka H, Fukuda Y, Ryo K, Emoto N, Kawai H, et al. Efficacy of right ventricular free-wall longitudinal speckle-tracking strain for predicting long-term outcome in patients with pulmonary hypertension. Circ J. 2013;77(3):756-63.

33. Park JH, Park MM, Farha S, Sharp J, Lundgrin E, Comhair S, et al. Impaired Global Right Ventricular Longitudinal Strain Predicts Long-Term Adverse Outcomes in Patients with Pulmonary Arterial Hypertension. J Cardiovasc Ultrasound. 2015;23(2):91-9.

34. Fine NM, Chen L, Bastiansen PM, Frantz RP, Pellikka PA, Oh JK, et al. Outcome prediction by quantitative right ventricular function assessment in 575 subjects evaluated for pulmonary hypertension. Circ Cardiovasc Imaging. 2013;6(5):711-21.

35. Haeck ML, Scherptong RW, Marsan NA, Holman ER, Schalij MJ, Bax JJ, et al. Prognostic value of right ventricular longitudinal peak systolic strain in patients with pulmonary hypertension. Circ Cardiovasc Imaging. 2012;5(5):628-36.

36. Hardegree EL, Sachdev A, Villarraga HR, Frantz RP, McGoon MD, Kushwaha SS, et al. Role of serial quantitative assessment of right ventricular function by strain in pulmonary arterial hypertension. Am J Cardiol. 2013;111(1):143-8.

37. Hulshof HG, Eijsvogels TMH, Kleinnibbelink G, van Dijk AP, George KP, Oxborough DL, et al. Prognostic value of right ventricular longitudinal strain in patients with pulmonary hypertension: a systematic review and meta-analysis. Eur Heart J Cardiovasc Imaging. 2019;20(4):475-84.

38. Bannehr M, Kahn U, Liebchen J, Okamoto M, Hähnel V, Georgi C, et al. Right Ventricular Longitudinal Strain Predicts Survival in Patients With Functional Tricuspid Regurgitation. Can J Cardiol. 2021;37(7):1086-93.

39. Prihadi EA, van der Bijl P, Dietz M, Abou R, Vollema EM, Marsan NA, et al. Prognostic Implications of Right Ventricular Free Wall Longitudinal Strain in Patients With Significant Functional Tricuspid Regurgitation. Circ Cardiovasc Imaging. 2019;12(3):e008666.

40. Romano S, Dell'atti D, Judd RM, Kim RJ, Weinsaft JW, Kim J, et al. Prognostic Value of Feature-Tracking Right Ventricular Longitudinal Strain in Severe Functional Tricuspid Regurgitation: A Multicenter Study. JACC Cardiovasc Imaging. 2021;14(8):1561-8.
